# Control of COVID-19 in Australia through quarantine: the role of special health accommodation (SHA) in New South Wales, Australia

**DOI:** 10.1186/s12889-021-10244-7

**Published:** 2021-01-27

**Authors:** Penelope Fotheringham, Teresa Anderson, Miranda Shaw, Joseph Jewitt, Hannah Storey, Owen Hutchings, Jason Cartwright, Leena Gupta

**Affiliations:** 1 Sydney Local Health District (SLHD), Camperdown, Australia; 2 Healthcare Australia, Osborne Park, Australia

**Keywords:** Quarantine, Public health, Health hotel, COVID-19, Virtual hospital

## Abstract

**Background:**

The first COVID-19 cases were diagnosed in Australia on 25 January 2020. Initial epidiemiology showed that the majority of cases were in returned travellers from overseas. One aspect of Public Health response was to introduce compulsory 14 day quarantine for all travellers returning to New South Wales (NSW) by air or sea in Special Health Accommodation (SHA). We aim to outline the establishment of a specialised health quarantine accommodation service in the context of the COVID-19 pandemic, and describe the first month of COVID-19 screening.

**Methods:**

The SHA was established with a comprehensive governance structure, remote clinical management through Royal Prince Alfred Virtual Hospital (**rpa**virtual) and site management with health care workers, NSW Police and accommodation staff.

**Results:**

From 29 March to 29 April 2020, 373 returning travellers were admitted to the SHA from Sydney Airport. 88 (26.1%) of those swabbed were positive for SARS-CoV 2. The day of diagnosis of COVID-19 varied from Day 1 to Day 13, with 63.6% (*n* = 56) of these in the first week of quarantine. 50% of the people in the SHA were referred to **rpa**virtual for ongoing clinical management. Seven people required admission to hospital for ongoing clinical care.

**Conclusion:**

The Public Health response to COVID-19 in Australia included early and increased case detection through testing, tracing of contacts of confirmed cases, social distancing and prohibition of gatherings. In addition to these measures, the introduction of mandated quarantine for travellers to Australia was integral to the successful containment of COVID-19 in NSW and Australia through the prevention of transmission locally and interstate from returning travellers.

The first COVID-19 cases in Australia were diagnosed on 25 January 2020 in Victoria and NSW. By the end of March 2020 there were 2182 confirmed cases in NSW from 217,030 tests performed [1]. Initial epidemiology showed that the majority of confirmed cases were amongst recent arrivals from overseas and their contacts [2]. Due to this a major feature of Australia’s public health response to SARS-CoV-2 has been to restrict population movements, especially through international travel, with the use of legislated quarantine. Quarantine refers to the separation of those that have been exposed to a disease but are not yet symptomatic from others who are susceptible to the disease (close contacts). Isolation refers to the process where people who have been diagnosed with the disease (cases) are separated from those who are not infected. Widespread use of quarantine in Australia has not occurred since the Influenza pandemic of 1918, when Maritime quarantine commenced on 17 October 1918 prior to the first cases of influenza in January 1919 [3, 4].

In addition to quarantine, other public health measures to control COVID-19 have been implemented such as early and increased case detection through testing, tracing of contacts of confirmed cases, social distancing and prohibition of gatherings [5, 6]. Australian modelling studies showed that in order to mitigate disease spread in Australia, isolation, quarantine and social distancing were required to ensure that Intensive Care Units (ICU) and the hospital system more broadly were not overwhelmed by a rapid increase in COVID-19 cases [7–9].

.In countries that introduced quarantine as part of their containment strategy, it was recognised that there was a need for a health facility that could manage non-COVID related medical illnesses in those subject to quarantine, as well as ensuring appropriate implementation of the other public health measures required for containment [10–12]. These measures have been combined in the Special Health Accommodation (SHA), a service that provides a quarantine environment with the ability to test suspected cases, clinically manage suspected and confirmed cases and cohort individuals in a supportive setting. The aim of this paper is to describe the new approach to quarantine developed in the SHA, the first Australian “health hotel” for COVID-19-related isolation, quarantine and clinical management, and to document the initial occupancy and COVID-19 management.

## Quarantine in NSW, Australia

The NSW Government issued multiple Orders under the Public Health Act regarding COVID-19 restricting movement of returned travellers. The first Order, on 17 March, 2020, mandated self-quarantine for 14 days for any returned travellers to NSW [13]. Australian borders were closed to non-citizens and residents from 9 pm AEDT Friday, 20 March, and a further Order, on 29 March, enforced mandatory quarantine in a quarantine or medical facility for 14 days after arriving in NSW by air or sea [14, 15].

The Sydney Local Health District (SLHD) had established an accommodation service for community members who were required to isolate or quarantine due to COVID-19 in February 2020. However, when the mandatory quarantine order was enacted on 29 March, SLHD rapidly extended the existing health accommodation service to establish the SHA, a unique service designed to support those affected by the NSW public health response to COVID-19. The existing service had been responsible for housing the majority of displaced people from the community in NSW who could not effectively quarantine and isolate following earlier COVID-19 Public Health Orders [16]. It was broadened to include those now subject to the Public Health Orders (COVID-19 Air Transportation Quarantine and COVID-19 Maritime Quarantine) that mandated quarantine in a quarantine or medical facility for returned travellers.

## Methods

### Establishment of the NSW special health accommodation service

**Fig. 1:**
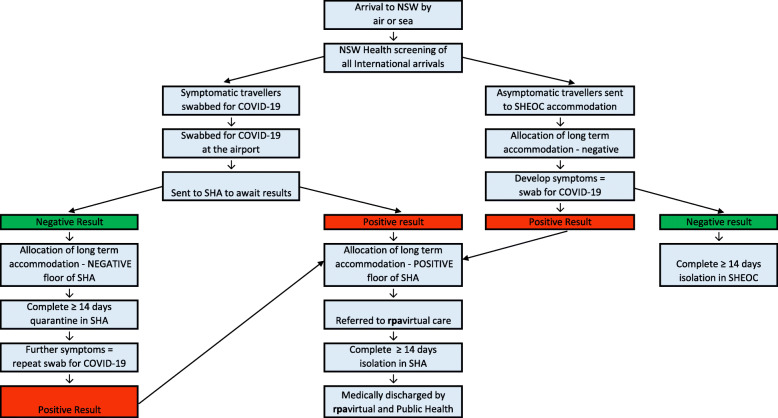
Referral flow chart for Returning International Travellers to NSW Special Health Accommodation

Since 29 March 2020 returning travellers to NSW have been assessed and triaged for allocation to appropriate accommodation by trained NSW Health officials. Returned travellers are triaged for accommodation according to their state of health on arrival. Asymptomatic returned travellers are managed in accommodation for quarantine that is run by NSW Police and supported by the NSW State Health Emergency Operations Centre (SHEOC) and Health Care Australia. Returning travellers symptomatic of an influenza-like-illness (ILI) are tested for COVID-19 with a nasopharyngeal swab through polymerase chain reaction (PCR) to detect the SARS-CoV-2 nucleic acid (RNA). The triage process for allocation to these services from arrival is outlined in Fig. 1.

The SHA provides comprehensive health care services to people that either have COVID-19, are at risk of having COVID-19 or who have complex health needs that are not appropriate for management in the Police managed Quarantine Hotels. The SHA also cares for people from the community who are COVID-19 positive or close contacts of people who are COVID_19 positive and are unable to self-isolate at home. Patients are transferred to Royal Prince Alfred (RPA) Hospital if their condition deteriorates and they are unable to be safely managed in the SHA.

By mid-April the accommodation comprised of five repurposed hotel facilities that were negotiated for use by the SLHD. On arrival at the SHA the incoming travellers are met by a nurse in personal protective equipment (PPE) and are then escorted and orientated to their accommodation. There is a 356 room capacity with single, double and family configured accommodation options, specifically designed to ensure that the needs of different traveller groups can be appropriately managed.

All travellers quarantined in the SHA are considered virtual patients with an electronic medical record, and their care is managed by health professionals. There is strict physical separation of the patient cohorts on different floors of the service. Stringent infection control protocols and measures are in place with Personal Protective Equipment (PPE) used for all staff and patient encounters due to the high risk of development of COVID-19 of those quarantined in the accommodation, and for the management of the confirmed COVID-19 cases completing their isolation. Both contact and droplet precautions and aerosol precautions are used in the SHA, according to the infection control protocol.

This service is unique in the state and is able to house any person who enters Australia through NSW. This includes Australian citizens, permanent residents and overseas residents who are returning to Australia. Anyone admitted to the SHA is required to be ambulant, have the capacity to manage their own medications and any pre-existing conditions should be suitable for remote medical management. If any person becomes significantly unwell during their quarantine period, they are remotely assessed by the **rpa**virtual medical team and then transferred by ambulance to RPA Hospital Emergency Department if they require a higher level of clinical care.

### Governance structure

**Fig. 2:**
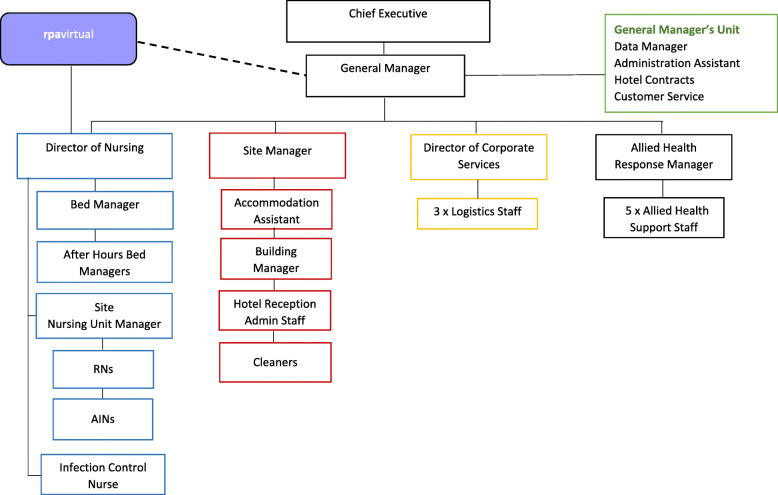
Clinical Governance Structure of the Special Health Accommodation

The SLHD established a comprehensive governance structure (Fig. 2) for the SHA to ensure both medical and Public Health matters were managed appropriately. The managers at each site report to the General Manager of the SHA, who is responsible for the planning, directing and ongoing management of the operations. Medical governance is provided by the A/Clinical Director of **rpa**virtual, the 24/7 virtual hospital service in the SLHD. There is a high level of oversight from the Chief Executive (CE) of the SLHD regarding decisions made within the service. This includes advice regarding appropriate selection and allocation of accommodation, initial investigations for those that become medically unstable and for all patient discharges. Routine data collection of any returned traveller is managed through the SLHD Executive to ensure that appropriate systems review and quality assurance reviews can take place.

### Site management

As outlined in Fig. 2, a variety of health care workers are on site 24/7 in the SHA. Registered nurses (RN) and assistants-in-nursing (AIN) are present 24/7 on site for health care needs. Additional staff include: the Site Manager, Accommodation Assistant, receptionist, cleaning staff and the building manager. Allied health staff on site consist of dieticians, social workers, psychologists, physiotherapists and speech pathology staff. They are able to cater to the varied needs of the residents as they arise.

All staff wear appropriate PPE and their designation is indicated by a sticker so that all patients are aware of the staff they may interact with. Any staff that enter the site answer screening questions for COVID-19 that include symptoms and possible exposures as per the NSW Health Guidelines [17]. Staff who are symptomatic, have been to an identified COVID-19 hotspot or are a close contact of a COVID-19 case are not allowed entry and are required to undergo risk assessment and COVID-19 testing prior to returning to work. NSW Police and security staff monitor the perimeter of the facilities to protect staff, the patients and the community in the event of patients not complying with quarantine requirements. They do not routinely enter the facility unless requested and supervised by clinical staff in the event of an emergency.

### Clinical management

Clinical management of patients who are positive for COVID-19 occurs through **rpa**virtual and the SLHD Public Health Unit (PHU). **rpa**virtual is a virtual hospital operating from SLHD’s RPA Hospital campus. This service provides remote, around-the-clock care for COVID-19 and other patients. It is supported by RPA Hospital specialists with an on-call roster for all sub-specialties. The joint management by clinical teams and the PHU is to ensure that patients’ clinical needs are met and that appropriate Public Health Guidelines for isolation and clearance testing are applied to each case following their diagnosis. **rpa**virtual supports both the physical and mental health of these patients, and also provides advice for their families. Soon after implementation of virtual care for COVID-19 patients and in response to the levels of stress and anxiety displayed by patients in SHA quarantine, **rpa**virtual introduced a roster of psychologists and social workers. If any patient in the accommodation service becomes medically unstable and requires an escalation of their care this is co-ordinated with the **rpa**virtual Clinical Director, the PHU, NSW Ambulance service and the Emergency Department of Royal Prince Alfred Hospital.

Welfare checks for all patients in the SHA are undertaken twice daily by qualified nursing staff. These take place through phone call and text messaging, dependent upon the preference of the patient. If there is no reply from the patient then this is escalated to an in-person room check to ensure they are well. Adjustment to the loss of individual autonomy and uncertainty around their individual health has meant that a large range of issues are managed by these staff. The rare episodes of aggressive behaviour are escalated through security as required, and questioning of quarantine requirements is locally managed and then escalated to the Public Health Unit as required.

### Discharge

Discharge management of all persons in the SHA occurs in conjunction with the PHU. Each person’s individual circumstances are reviewed by **rpa**virtual, the PHU and the SLHD Executive team to confirm they have met the current recommendations in the COVID-19 Series of National Guidelines and that in approving discharge relevant Public Health Orders are followed [18]. For families with discordant test results, a discharge process is worked out with the family members, to ensure that the case has fulfilled criteria for clearance, and any exposed family members undergo the appropriate 14 day quarantine from their last exposure to the case. The evolution of the discharge process has involved communications with the NSW Ministry of Health and other jurisdictions within Australia, as many people in the SHA do not live within the Sydney Metropolitan area. Discharge planning has required co-ordination with both clinical and public health services in travellers’ relevant jurisdictions for them to be able to return to their homes given the current varying travel restrictions and Public Health Orders that exist within Australia.

## Results

### Demographics

Data reported represent the accommodation occupancy from 29 March 2020 to 29 April 2020. The total number accommodated in the SHA during this time period was 520, with 464 patients returned travellers, and 56 community patients from the SLHD. Community patients in the SHA consisted of people from within the SLHD and those from other sources such as cruise ships. The median age of patients in the SHA service was 35, ranging from 2 months to 86 years. Forty-six patients were children aged less than 18 years. There was an even distribution of gender with 182 female and 191 male patients. Returning travellers came from 5 international regions. The majority returned from North America 34% (*n* = 128) and Europe 30% (*n* = 113). In this timeframe cumulatively 9661 returned travellers were resident in the accommodation service managed by SHEOC.

### Person flow

A total of 373 people were admitted to the SHA directly from the airport. Of these 337 were symptomatic and tested for COVID-19, the remaining 36 were accompanying family members. From those who were swabbed 88 (26.1%) were positive for SARS-CoV 2, representing 0.87% of the total 10,034 returning air travellers to NSW in this time period. During the same period 902 people of the total 9661, (9.3% of occupancy) in the SHEOC Accommodation were swabbed and 76 people were transferred from SHEOC Accommodation to the SHA during their 14-day quarantine period due to need for remote medical care or a positive COVID-19 swab result. Of all the positive cases in the SHA the day of positive diagnosis varied from Day 1 to Day 13 of their quarantine period. 63.6% (*n* = 56) of these were diagnosed as positive in the first week of quarantine.

Fifty per-cent of people in SHA were referred to **rpa**virtual for ongoing clinical care, whether due to their positive status, or the need for other medical or allied health assistance. Seven of the patients who tested positive for COVID-19 became medically unstable and required admission to RPA Hospital for ongoing medical care. The distribution of positive results by arrival location is shown in Table 1.

**Table 1:** COVID-19 status in SHA by region of origin 29 March – 29 April 2020

Region of origin	COVID-19 Status			
	Negative	Positive	Not swabbed	Total
	*n*=250	*n*=87	*n*=36	*n*= 373
**Asia**	**29**	**7**	**7**	**43**
**Europe**	**73**	**27**	**27**	**127**
**North America**	**91**	**33**	**33**	**157**
**Oceania**	**41**	**1**	**1**	**43**
**South America**	**16**	**19**	**19**	**54**

During this time frame 284 of the 373 residents were discharged from the accommodation. The average length of stay in the SHA was 14.86 days (not including patients exempt from quarantine in NSW). The total quarantine length in any accommodation for patients was 16.17 days (including SHEOC accommodation stay). The average length of stay for the 72 discharged positive patients up to 30 April 2020 was 19.21 days (14–28 days).

The discharge address location for the patients varied. 58.4% (*n* = 218) returned to a NSW address and the rest to interstate addresses with the majority to Queensland 19% (*n* = 70) and 9% (*n* = 32) from Victoria.

## Incidents

An estimated 20% of patients reported concerns about their quarantine period to the SHA management. There were a total of 29 formal incidents notified through the internal SLHD system in this time – 14 complaints, 6 security, 5 accident/ occupational health and safety incidents, 3 aggression and 1 fall. On site police were involved in 5 incidents due to aggression from the patients that required escalation.

## Discussion

Australia’s COVID-19 pandemic response has posed unique challenges as well as opportunities for containment in the island nation. Australia has not faced a challenge to its population that has required this since the Influenza outbreak in 1918 [3] . Australia’s COVID-19 Public Health approach has been based on containment, as there is currently no effective vaccination or specific therapeutic options. Containment strategies such as quarantine, isolation, early case detection through testing and contact tracing, restriction of gatherings and implementation of social distancing are aimed at reducing person to person transmission as well as fomite transfer [19]. Modelling data which predicted the potential numbers of cases, deaths and impact on health service capacity informed the decisions to implement strong containment strategies and in particular, restrictive border measures, first with restriction of travel from China and then more broadly when analysis of transmission identified travel from other countries and cruise ships as factors introducing the epidemic in Australia [20–22].

The establishment of the SHA meant that quarantine isolation and management of suspected and confirmed cases, as well as management of close contacts could be comprehensively carried out for the returned traveller group. In NSW prior to the introduction of self-isolation for all returning travellers, notified cases were rapidly increasing, with 58% of cases comprising of returned travellers or their contacts up to 30 April 2020. After the closure of Australia’s borders on 20 March 2020, there was a gradual decrease in overall case numbers that was further reduced by the introduction of mandatory hotel quarantine on 29 March. Community transmission was also shown to decrease after this time [13]. Thus quarantine and isolation of returned travellers was a vital part of the Public Health response in mitigating COVID-19 spread in NSW and Australia.

The demographics of the returning traveller group reflected that when the borders were closed many families were attempting to return home with their children, and that older travellers and overseas residents were also returning to Australia. The broad age range also influenced the need for a ‘health hotel’ service, as shown by 51% of returned travellers requiring referral to **rpa**virtual for some form of remote medical care, with less than half of these being related to SARS-CoV-2 infection.

The ability to monitor the health and wellbeing of the patients in the accommodation also meant that early case detection was optimised for an at-risk population. By April 292,020 Australia had performed 544,410 COVID 19 tests with 1.2% of these positive. This represents coverage of approximately 2.1% of the Australian population [23, 24]. Our findings showed that 0.87% of returned travellers in the first month of the SHA tested positive for SARS-CoV-2, consistent with the Australian positive rates. The availability of testing within the SHA and the accommodation facilities managed by SHEOC throughout the quarantine period meant that new cases could be identified, isolated in an appropriate facility and managed clinically, while preventing ensuring separation from any accompanying at risk family members. The wide range of date of detection found in the returned travellers highlights the need for routine testing at the beginning and end of their quarantine period. Isolation of confirmed cases of COVID-19 until they had been clinically cleared by **rpa**virtual and the PHU meant that both the needs of the individual and the community were managed. The small number of people that required in hospital care returned to the SHA after discharge for any ongoing quarantine or clearance requirements after their admission.

Establishing a health service with multiple stakeholders and an evolving public health policy environment “on the run” was not without difficulty. The requirement to mobilise and provide a quarantine accommodation service required rapid recruitment and organisational management from NSW Health, NSW Police and the SLHD. Co-ordination and consistency of the aims of the accommodation was needed to follow the appropriate Public Health Orders as well as providing a high standard of clinical care for any unwell patients. At times, issues were identified only when problems or challenges arose, requiring a flexible, dynamic approach to management.

The ability to cater for individual needs was bound by the legal responsibility to ensure that the Public Health Orders were appropriately applied to every individual in the service in order to protect the needs of the community. Compulsory quarantine and isolation ensured that the welfare of the community was prioritised, however balancing the needs of the individual and the community was challenging. The heterogeneity of the patients within the SHA and their varying needs required adaptability and daily review of all patients to ensure that their individual needs were met. The length of stay within the SHA varied considerably due to the timing of diagnosis, length of illness and recovery period that allowed clearance for discharge following the COVID-19 Series of National Guidelines [18]. Length of stay was also increased for family members that required extended quarantine after being exposed to a family member that subsequently swabbed positive for COVID-19.

This was one of the challenging aspects of introducing and managing the SHA. SHA management estimated that 20% of patients escalated concerns regarding their quarantine period, however it is likely the true number is at least 50% due to the frequency of patients raising concerns with onsite staff that were not required to be escalated to more senior managers. Compliance with quarantine in previous International examples has varied significantly, and the number of those questioning their quarantine period potentially reflects those that would not adhere appropriately in the community.

Understandably, individuals wished to advocate for themselves and their families regarding the conditions of their stay and these concerns needed to be adequately and sensitively addressed. According to the SHA management team, those that challenged their quarantine had four broad reasons for questioning their requirements to follow the Public Health Order. Unfortunately, many patients became verbally aggressive and emotional in this process.

- Poor understanding of risk for themselves and the community led to many people questioning why they needed to follow the Public Health Order, requiring further detailed discussion. The degree of illness experienced was influential - whether they were symptomatic and requiring a swab, negative or a case that was diagnosed as positive. Those that had mild symptoms seemed more likely to question the need to comply with the Public Health orders and to express dissatisfaction with their care.
- Some people understood the risks but formed the opinion that the risk did not apply to them.
- Some questioned the ethics and legality of the Public Health orders as they had been written and the application of them to their individual situation.
- Many people also found quarantine challenging to maintain due to their personal or family circumstances.

The importance of clear and consistent communication, and the provision of information has been shown to be important for the wellbeing of those adhering to quarantine [25]. When queries and concerns were raised, decision makers had the dual pressures of ensuring a timely and appropriate response for staff and patients, and navigating the potential ongoing implications of any decision made on the service and on the community. As the service has evolved, communication channels have become better understood and managed. The clear governance structure and delineation of roles within the management of the service allowed any issue that arose to be managed in a co-ordinated fashion, involving the General Manager of the service, the Public Health Unit, **rpa**virtual and the Chief Executive of the SLHD.

## Conclusion

We have described the evolution of a unique health and accommodation service that required rapid recruitment and organisational management from NSW Health, NSW Police, Health Care Australia and the SLHD. This occurred in response to the need to follow the appropriate Public Health Orders while providing a high standard of clinical care for unwell patients. The current Public Health orders that exist in NSW and around Australia restricting the freedom of movement of individuals have not been in place in living memory for the majority of the population.

The SHA is a novel and important demonstration of the application of the Public Health principle of quarantine in contributing to the containment of COVID-19 in NSW, Australia. The learning from this process of establishment and management of the service is invaluable for any future disease outbreak requiring widespread quarantine and isolation in the population.

Ongoing review of the SHA is being undertaken to ensure that improvements to the service can be made for patients, staff and management. Long term follow up of the patients is planned to evaluate the impact of the quarantine on the health and social circumstances of the patients and staff involved in the service.

## Data Availability

The datasets generated during and/or analysed during the current study are available in the figshare repository 10.6084/m9.figshare.12464072.

## Abbreviations

AIN: Assistant-in-nursing
ICU: Intensive Care Unit
NSW: New South Wales
PHU: Public Health Unit
PPE: Personal Protective Equipment
PCR: Polymerase chain reaction
RN: Registered nurse
RPA Hospital: Royal Prince Alfred Hospital
Rpavirtual: Royal Prince Alfred Virtual Hospital
SHA: Special Health Accommodation
SHEOC: State Health Emergency Operations Centre
SLHD: Sydney Local Health District
